# COVID-19 and Autoimmunity in Dermatology: A Moroccan Case Series and Literature Review

**DOI:** 10.7759/cureus.57587

**Published:** 2024-04-04

**Authors:** Fatimazahrae Benhayoun, Fouzia Hali, Fatima Zahra El Fatoiki, Soumiya Chiheb

**Affiliations:** 1 Dermatology, Centre Hospitalier Universitaire Ibn Rochd (CHU Ibn Rochd), Casablanca, MAR

**Keywords:** covid-19, toxidermia, autoimmunity, pathophysiological mechanisms, dermatological manifestations

## Abstract

Introduction: Since the beginning of the pandemic, many skin manifestations associated with COVID-19 have been reported. New reports show that COVID-19 can lead to autoimmune diseases (AIDs) and autoinflammatory diseases, especially dermatological.

Methods: A prospective study was conducted by the dermatology department of the Centre Hospitalier Universitaire Ibn Rochd (CHU Ibn Rochd) of Casablanca in Morocco since the beginning of the pandemic including 18 patients with COVID-19-related skin manifestations.

Results: Eighteen cases were collected with confirmed SARS-CoV-2 infection. The mean COVID score was 0.7. A percentage (94.44%) of the cases had general symptoms. Skin involvement was variable, mainly maculopapular rash (44.44%), purpura (27.77%), urticaria, varicelliform rash, necrotic lesions of the face, and pityriasis rosea Gibert (PRG)-like lesions. Mucosal involvement was found in 50%. Viral reactivation was found in 5.55%. Telogen effluvium was found in 22.22%. Moreover, AID was triggered by COVID-19: lupus (11.11%), associated with antiphospholipid syndrome (APL Sd) (5.55%), psoriasis (11.11%), alopecia, and pemphigus. Severe toxidermia was potentiated by SARS-CoV-2 infection (22.22%): Stevens-Johnson syndrome (Sd), acute generalized exanthematous pustulosis (APEG), and drug reaction with eosinophilia and systemic symptoms (DRESS).

Conclusion: The interest of this work is to report our experience during the COVID-19 pandemic to understand some pathophysiological mechanisms of its dermatological manifestations and to draw the attention of clinicians to the link of this infection with autoimmune and autoinflammatory diseases and toxidermia.

## Introduction

Since the beginning of the pandemic, numerous skin manifestations associated with COVID-19 have been reported in the literature. However, new reports show that COVID-19 can lead to autoimmune and autoinflammatory diseases, especially dermatological diseases. Here, we report the experience of the dermatology department of the Centre Hospitalier Universitaire Ibn Rochd (CHU Ibn Rochd) of Casablanca in Morocco during this pandemic.

## Materials and methods

This is a prospective study conducted by the dermatology department of the CHU Ibn Rochd of Casablanca since the beginning of the pandemic, including 18 patients with cutaneous manifestations related to COVID-19. 

Inclusion criteria

All patients with dermatological manifestations triggered by SARS-CoV-2 infection were included.

Exclusion criteria

Patients with dermatological lesions prior to SARS-CoV-2 infection were excluded.

Data were entered and analyzed using Microsoft Excel software (Microsoft Corporation, USA).

All patients were given informed consent prior to inclusion. The study was conducted in accordance with the principles of the Declaration of Helsinki and local ethical guidelines (Ethics Committee for Biomedical Research, Faculty of Medicine and Pharmacy, Casablanca, Morocco). Patients gave their consent for photos to be taken and for their data to be used. Patient anonymity was respected.

## Results

A total of 18 cases were collected (Table 1). SARS-CoV-2 infection was confirmed by positive reverse transcriptase-polymerase chain reaction (RT-PCR) in 15 cases (83.33%), chest CT in four cases (22.22%), and serology (IgM+/- IgG on blood sample) in three cases (16.66%). The sex ratio was 0.38. The mean age was 38.3 years. The COVID score ranged from 0.1 to 7 with an average of 0.69. The positive contact was found in 13 cases (72.22%). Seventeen cases (94.44%) presented general symptoms, mainly fever, which was present in 16 cases (88.88%).

**Table 1:** Demographic characteristics of COVID-19-infected patients (%).

Number of cases (N)	18 cases
Demographic characteristics	
Mean age (mean)	38.3 years old
Sex ratio	0.38
COVID-19 mean score (Mean)	0.69
Past history	
High blood pressure (%)	0%
Diabetes (%)	11.11%
Obesity (%)	55.55%
Smoking (%)	16.66%
Lung disease (%)	0%
MAI (%)	5.55%
Atopy (%)	5.55%
Pregnancy (%)	5.55%
Confirmation of COVID-19 infection (%)	100%
General signs (%)	94.44%

**Figure 1:**
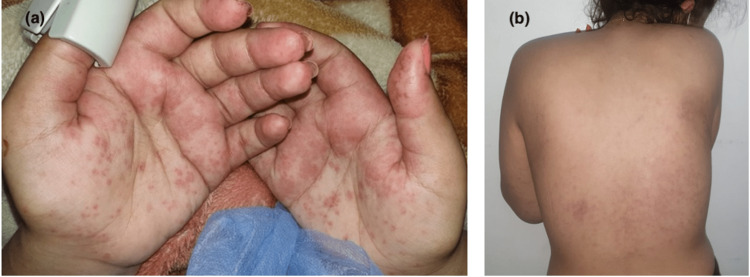
Palmar involvement (a) with maculo-papular exanthema (b)

**Figure 2:**
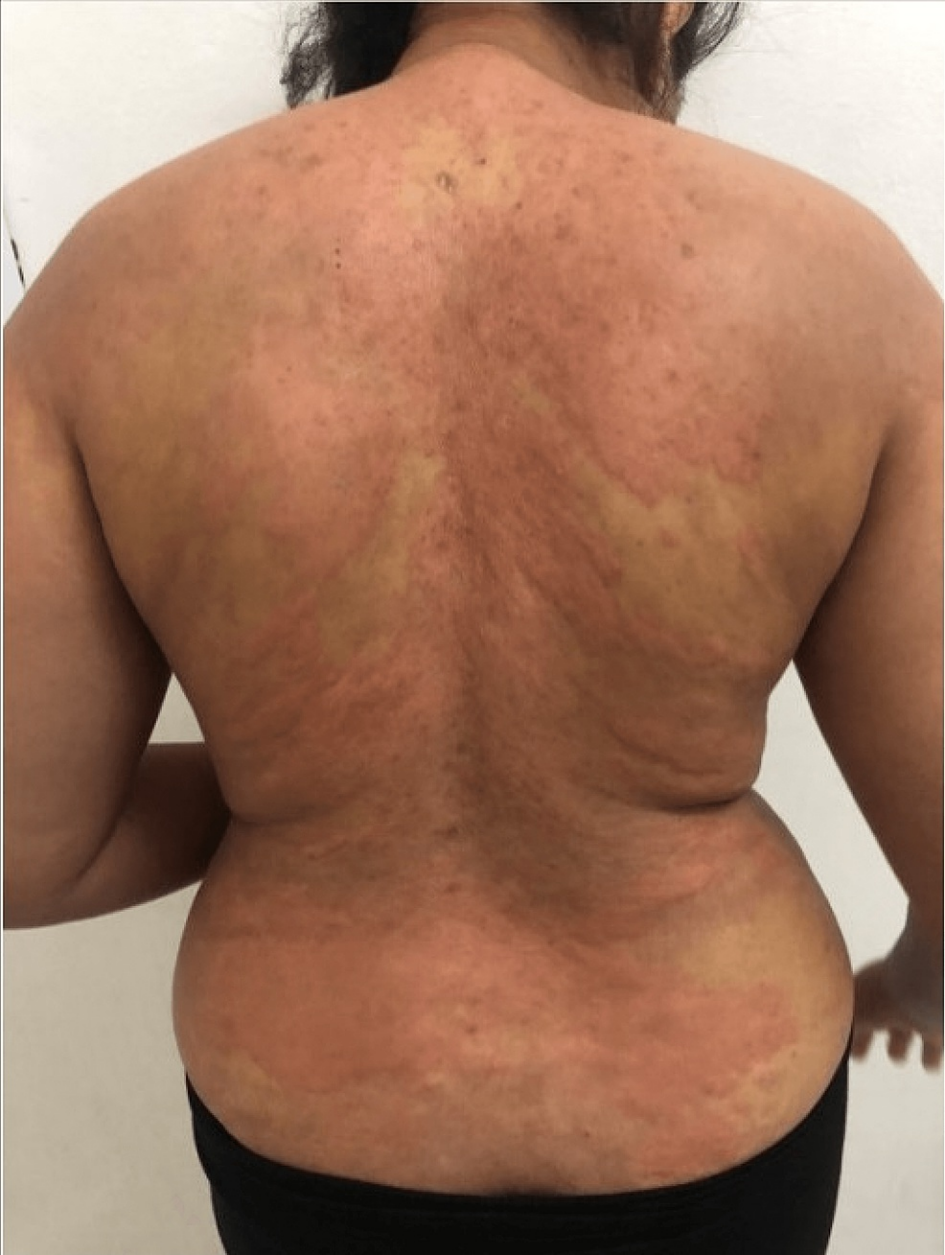
Urticarial rash

**Figure 3:**
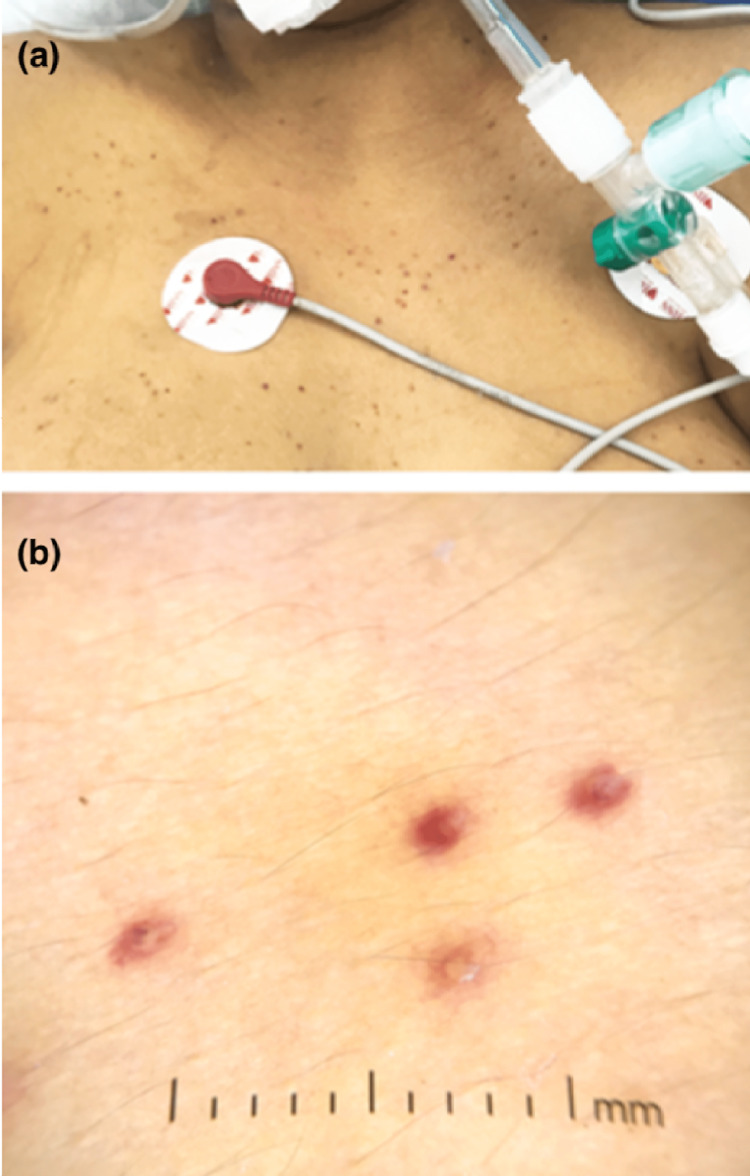
Varicelliform rash in a patient admitted to the ICU: clinical image (a) and dermoscopic image (b)

**Figure 4:**
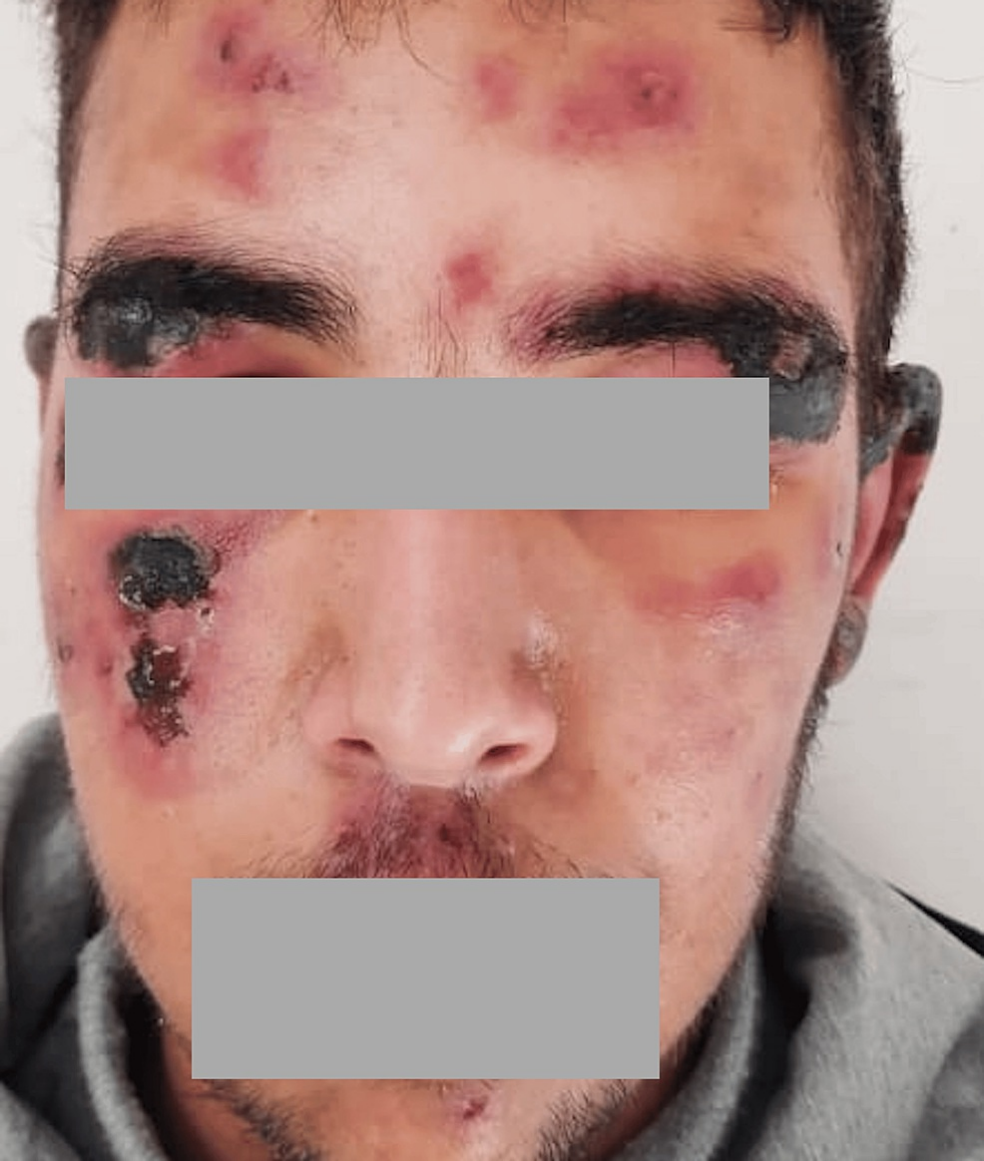
Purpuric and necrotic rash on the face

**Figure 5:**
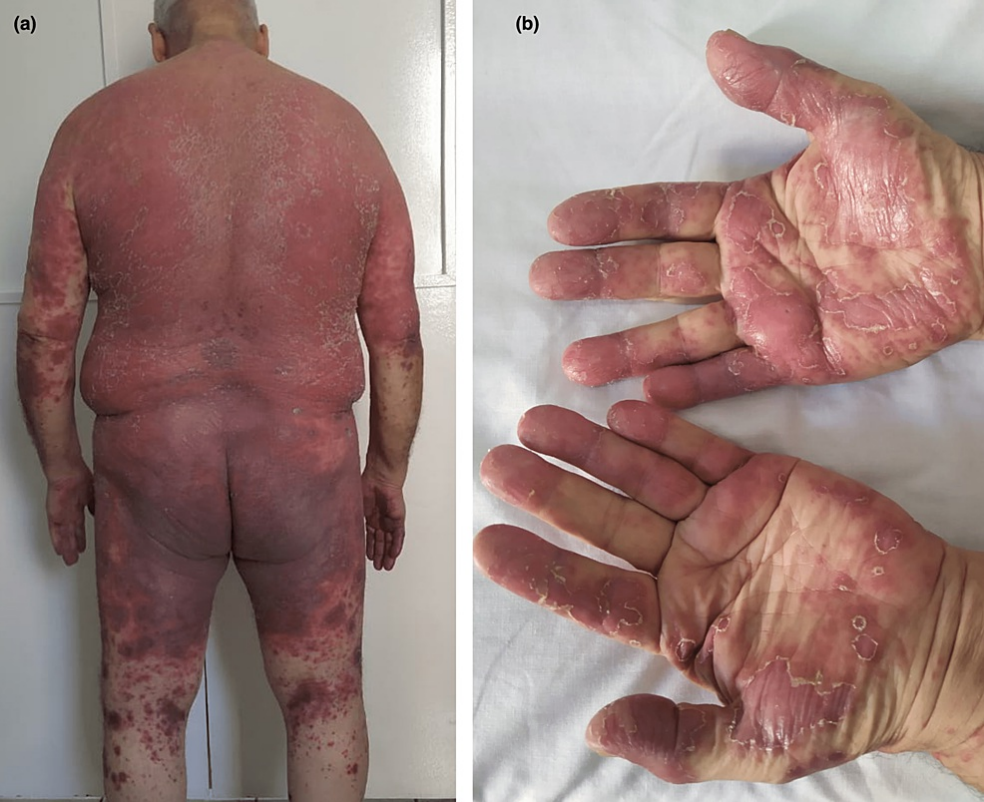
Erythematosquamous lesions and purpuric in some areas (a), with palmar involvement (b)

**Figure 6:**
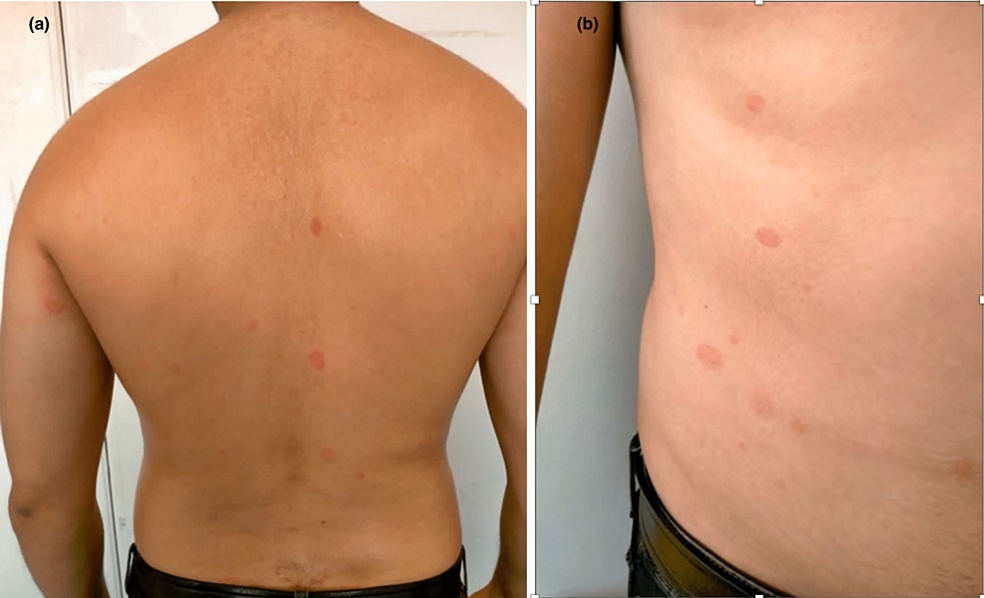
PRG-like lesions in the back (a) and abdomen (b) PRG: Pityriasis rosea Gibert

Skin involvement was variable (Table 2): maculopapular rash in eight cases (44.44%) (Figure 1), purpura in five cases (27.77%), and pustular rash in three cases (16.66%), followed by urticaria (Figure 2), varicelliform rash (Figure 3), necrotic lesions of the face (Figure 4), bullous lesions, erythematosquamous lesions (Figure 5), and Pityriasis rosea Gibert (PRG)-like lesions (Figure 6), each of which was present in one case (5.55%).

**Table 2:** Clinical characteristics of COVID-19-infected patients (%). PRG: Pityriasis rosea Gibert, DRESS: drug reaction with eosinophilia and systemic symptom, AGEP: acute generalized exanthematous pustulosis, APL: antiphospholipid syndrome

Number of cases (N)	18 cases
Dermatological manifestations	
Paraviral eruptions	
Maculo-papular rash (%)	44.44%
Urticarial rash (%)	5.55%
PRG-like (%)	5.55%
Vesicular/varicelliform rash (%)	5.55%
E. vasculitic	
Purpura (%)	27.77%
Necrosis (%)	5.55%
Pseudo-chilblains (%)	5.55%
Mucosal involvement: conjunctivitis, oral erosion (%)	77.77%
Viral reactivation: herpetic gingivostomatitis (%)	5.55%
Phanera: Telogen effluvium (%)	22.22%
Triggered autoimmune diseases	
Lupus (%)	11.11%
Sd APL (%)	5.55%
Psoriasis (%)	11.11%
Pemphigus (%)	5.55%
Alopecia areata (%)	5.55%
Potentiated toxidermia (%)	22.22%
Stevens-Johnson syndrome (%)	11.11%
AGEP (%)	5.55%
DRESS (%)	5.55%
Dermatological manifestations upon COVID-19 infection	
Revealing infection (%)	33.33%
During infection (%)	16.66%
After infection (%)	55.55%
Treatment of the infection	
Outpatient (%)	38.88%
Intensive care (%)	22.22%
Evolution	
Recovery (%)	83.33%
Death (%)	16.66%

**Figure 7:**
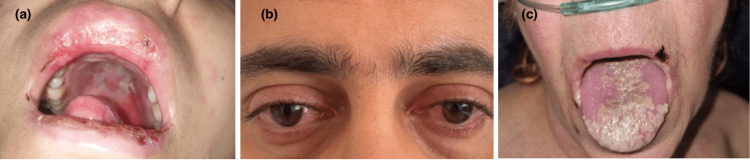
(a) Erosive cheilitis with an erosion of the palate, (b) conjunctivitis, and (c) herpetic gingivostomatitis

Mucosal involvement (Figure 7) was observed in 14 cases (77.77%): oral erosions in eight cases (44.44%), conjunctivitis in five cases (27.77%), and genital erosion in one case (5.55%). Viral reactivation was found in one case (5.55%), of type herpetic gingivostomatitis. This condition was most often found in patients with moderate to severe COVID-19 infection, accounting for 10 cases (55.55% of cases), with four cases requiring care in an intensive care unit (40%). It should be noted that there was no association between anosmia and/or ageusia and oral mucosal involvement.

**Figure 8:**
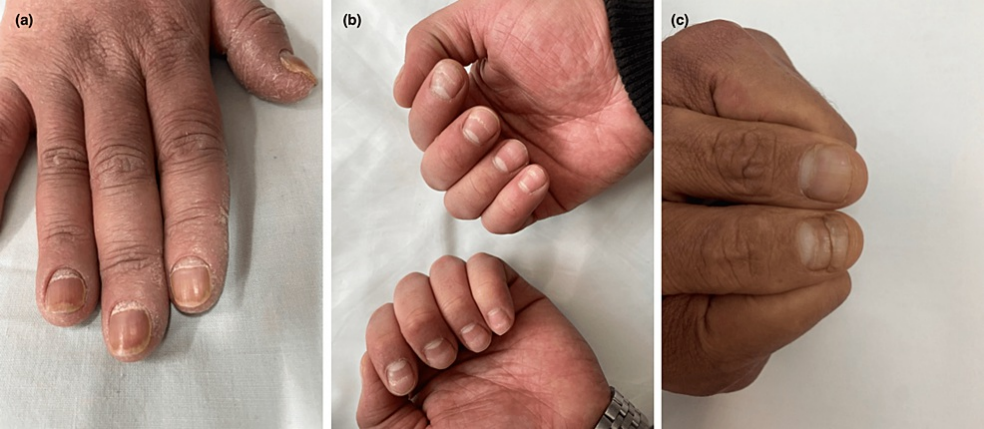
Onychomadesis in patients with COVID-19 infection (a, b, c)

Telogenous effluvium was found in four cases (22.22%), associated with fever in all our patients. Nail involvement was found in three cases (16.66%), mainly with onychomadesis (Figure 8).

**Figure 9:**
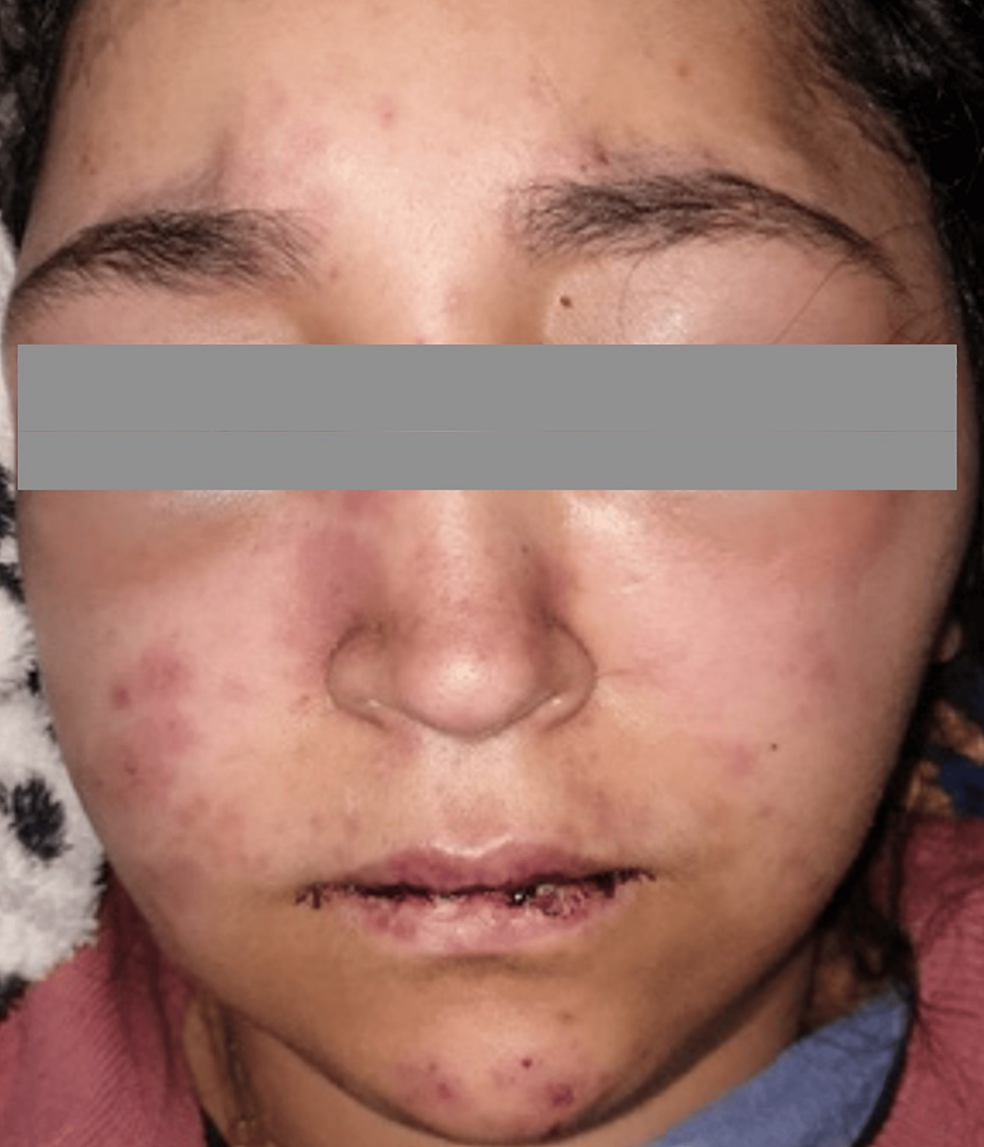
Systemic lupus erythematosus complicated by macrophagic activation syndrome

**Figure 10:**
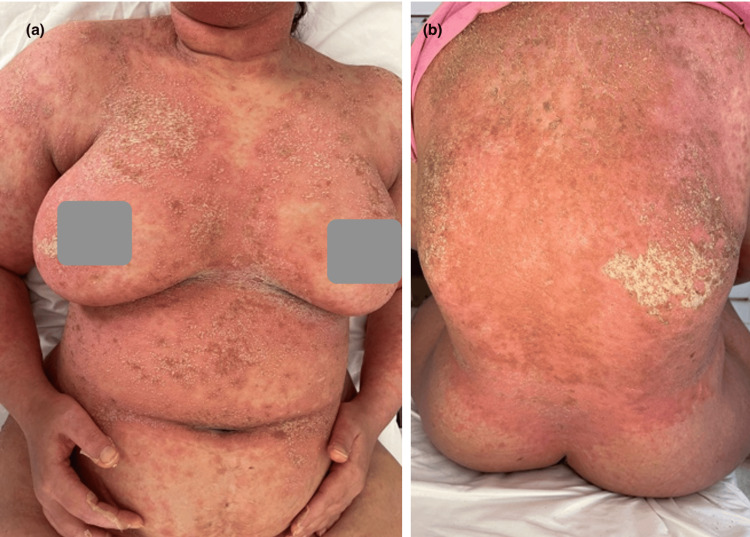
De novo pustular psoriasis in a patient in the trunk (a) and back (b)

**Figure 11:**
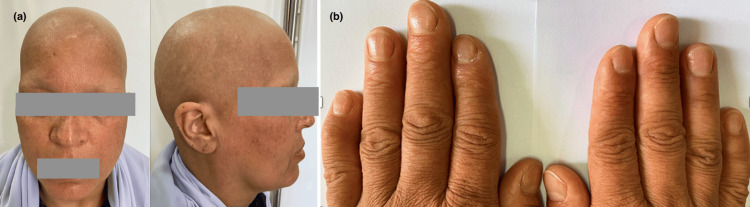
Universal alopecia (a) with nail involvement (b)

Furthermore, autoimmune diseases were triggered by COVID-19: systemic lupus erythematosus (SLE) in two patients (11.11%), associated with antiphospholipid syndrome (APL Sd) in one patient (5.55%), and complicated by macrophagic activation syndrome (SAM) in another patient (Figure 9), psoriasis in two patients (11, 11%) (pustular and vulgar psoriasis in 5.55% each) (Figure 10), universal alopecia in one case (5.55%) (Figure 11) (which relapsed after COVID-19 vaccination despite continued treatment), and pemphigus vulgaris in 1 case (5.55%). The majority of these patients did not have an autoimmune background.

**Figure 12:**
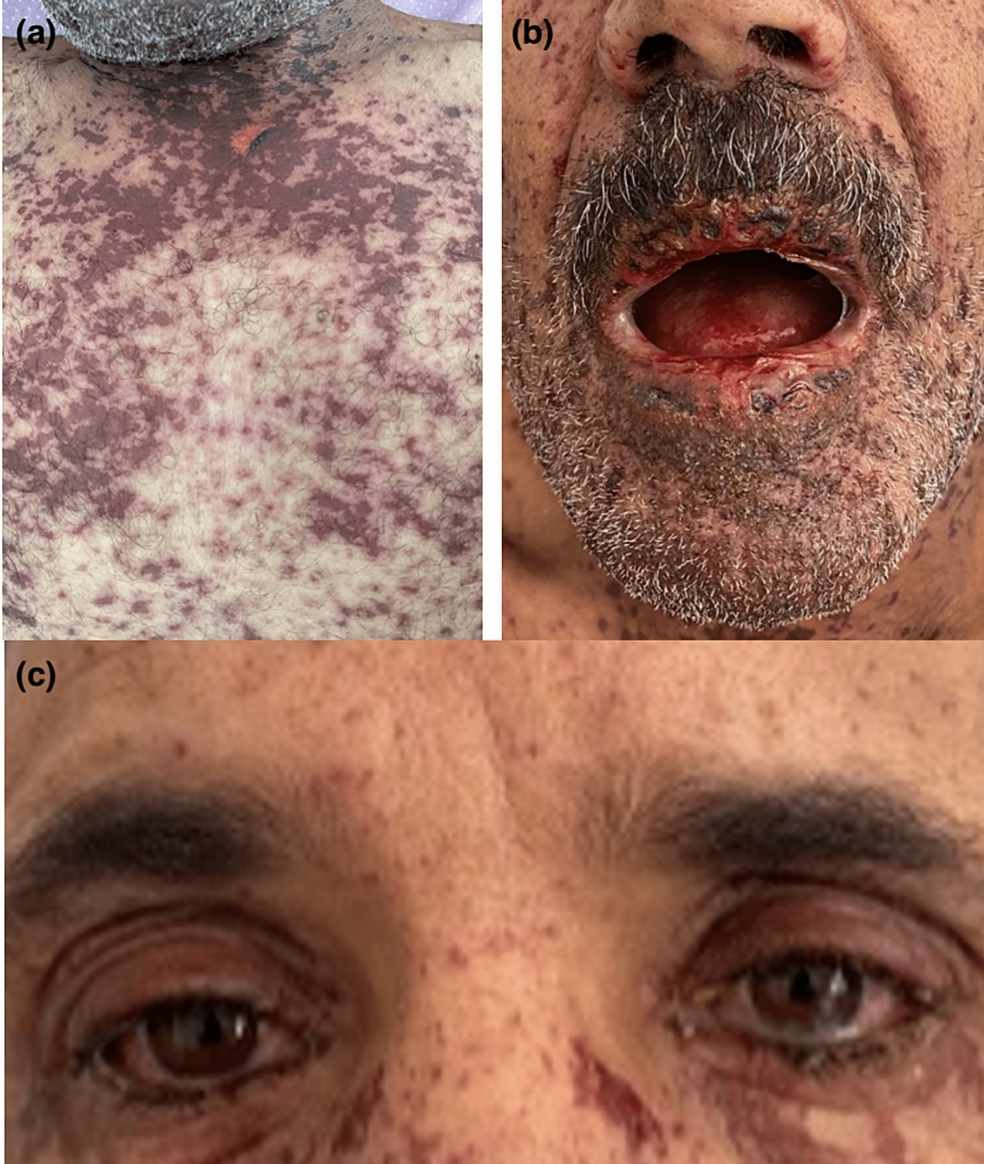
Stevens-Johnson syndrome, with Nikolsky + (a), and mucosal involvement (b, c), potentiated by COVID-19 infection

**Figure 13:**
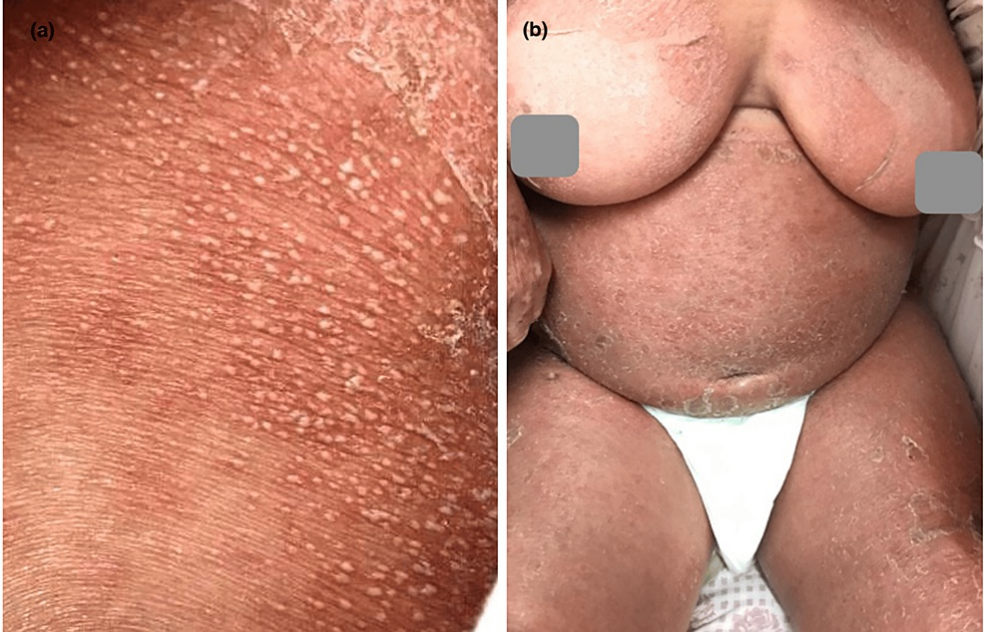
APEG potentiated by COVID-19 infection with pustular lesions in the back (a) and trunk (b)

Toxidermia was potentiated by SARS-CoV-2 infection in four cases (22.22%): Stevens-Johnson syndrome in two cases (11.11%) (Figure 12), acute generalized exanthematous pustulosis (AGEP) (Figure 13), and drug reaction with eosinophilia and systemic symptom (DRESS) syndrome in one case (5.55%), one of which progressed to Stevens-Johnson syndrome despite the discontinuation of all medications.

The mean time from symptom onset to dermatologic manifestations was 21.55 days. Dermatological involvement revealed SARS-CoV-2 infection in six cases (33.33%). Skin biopsy was performed in 11 cases (61.11%). Four cases (22.22%) required management in an intensive care setting. Three cases died (16.66%).

## Discussion

Dermatological involvement in COVID-19 remains rare with a worldwide incidence of 1-2%. This could be explained by the low proportion of ACE2 receptors of the virus in the skin compared to the alveoli and also by an under-reporting of cases of cutaneous manifestations due to their lesser severity [1].

The cutaneous manifestations have been classified into three groups: paravarial eruptions (maculopapular exanthema, urticaria, and PRG-like rash), varicelliform eruptions, and vasculitic eruptions (pseudoengelures, purpura, livedo, and necrosis), with the possibility of lesion polymorphism [2,3].

Mucosal involvement is rarely described and can be aphthoid lesions, herpetiform lesions, oral erosions, and conjunctivitis. It is associated with skin involvement in 29% of cases. Anosmia and ageusia are often present. Mucosal involvement has been associated with the severity of COVID-19 infection [4,5].

Telogen effluvium post-COVID has also been described in the literature, most often associated with febrile forms of infection. However, asymptomatic forms may exist, hence the interest in screening for COVID-19 infection in the face of an unexplained acute telogen effluvium. Its pathophysiology has been explained by the transition from the anagen to the telogen phase of the hair cycle, triggered by proinflammatory cytokines responsible for excessive hair loss [6].

Nail involvement appears late in the fourth to fifth month after infection, probably due to the nail matrix and vascular lesions triggered by complement deposition induced by SARS-CoV-2. Microvascular disorders were found: enlarged and/or sinuous capillaries, reduced density, microhemorrhages, and microthromboses. COVID-toe or COVID-finger with pernio-like lesions has been reported. Acral gangrene has been correlated with the severity of infection with multisystem inflammation, such as the peri-nail scaling observed in Kawasaki-like (MIS-C). Other nail abnormalities have been reported, such as nail dystrophy (line of beauty, leukonychia, onychomadesis, and onycholysis) and chromonychia (red half-moon nail and orange distal staining) [7,8].

Furthermore, autoimmune diseases can be triggered by COVID-19 in genetically predisposed patients, following the activation of an aberrant immune response by the cytokine cascade (TNF-α, IL-6, IL-1β, IL-17, and IL-18) induced by SARS-CoV-2 [9] (Table 3).

**Table 3:** Case reports describing COVID-19 as a trigger of dermatologic autoimmune disease

Authors	Autoimmune disease	Age and sex	Skin lesions	Delay
Slimani et al. [10]	LES with Sd APL	F, 23 years old	Papular lesions « varicella-like »	13 days after COVID-19
Hali et al. [11]	LES with SAM	F, 25 years old	Maculopapular exanthema, palmoplantar involvement, periorbital edema, infiltrated purpura, oral mucosa involvement	Concomitant evolution
Zamani et al. [12]	LES	M, 43 years old	Urticaria	4 weeks after COVID-19
Bonometti et al. [13]	LES	F, 85 years old	Edema, fingertips, and lower limb cyanosis	-----
Severino et al. [14]	Morphea	F, 62 years old	White sclerotic lesions with red halo (lilac ring) on the trunk	While recovering from COVID-19
Capalbo et al. [15]	Alopecia areata	M, 38 years old	Some alopecia patches in the beard area	1 month after COVID-19
Rossi et al. [16]	Alopecia areata	F, 29 years old	Progressive hair loss with a patchy pattern in the vertex and parietal regions	1 month after COVID-19
Sgubbi et al. [17]	Alopecia areata	F, 54 years old	Hair loss with a patchy pattern in the temporoparietal	2 months after COVID-19
Fivenson et al. [18]	Alopecia universalis	F, 56 years old	Rapidly progressive hair loss causing loss of total body hair	2 months after COVID-19
Mathieu et al. [19]	Pustular psoriasis	F, 62 years old	Blisters on the palms of the hands spreading to the forearms, trunk, and scalp	2 weeks after COVID-19
Dadras et al. [20]	Pustular psoriasis	M, 60 years old	Extensive patch and pustular erythematous	26 days after COVID-19
Lotfi et al. [21]	Morphea	F, 57 years old	Sclerotic skin, arthralgia	While recovering from COVID-19

Toxidermia can also be potentiated by SARS-CoV-2 infection via complex immune reactivations, even if prior sensitization is lacking, in genetically predisposed subjects. Some drugs can induce an exaggerated inflammatory reaction that will join the viral immune reaction causing the virus-drug synergy. This synergy between drug and cytokine storm triggered by COVID-19, mainly TNF α, IFN-gamma, LT CD 8+, and Th17 deregulation, can induce a hypersensitivity reaction to the drugs involving toxidermia. However, a SARS-CoV-2 infection must be suspected and looked for in front of any toxidermia with incompatible occurrence mode (delay) and/or severe evolution, as in the case of our patient hospitalized at our department for the management of a DRESS syndrome, which turned thereafter to a Stevens-Johnson syndrome in spite of the stop of all medication [22-26].

The limitation of our work is the small size of our sample, which can be explained by the under-reporting of cases of cutaneous manifestations given the lesser severity compared to other diseases, particularly pulmonary, since we only received severe dermatological diseases, especially autoimmune disorders. However, the importance of our study lies in the fact that it highlights the frequency of autoimmune disorders in our Moroccan context triggered by COVID-19 (genetic predisposition).

## Conclusions

The interest of this work is to report our experience during the COVID-19 pandemic to understand some pathophysiological mechanisms of its dermatological manifestations and to draw the attention of clinicians to the link of this infection with autoimmune and autoinflammatory diseases, as well as toxidermia.

COVID-19 can affect various organ systems to varying degrees, including dermatological. It is responsible for a wide range of cutaneous signs, with a complex spectrum, and various classifications. Indeed, SARS-CoV-2 may act as a trigger for the development of autoimmune dysregulation in genetically predisposed individuals, as well as potentiating the development of toxidermia via complex immune reactivations.
